# The DNA methyltransferase inhibitor, guadecitabine, targets tumor-induced myelopoiesis and recovers T cell activity to slow tumor growth in combination with adoptive immunotherapy in a mouse model of breast cancer

**DOI:** 10.1186/s12865-020-0337-5

**Published:** 2020-02-27

**Authors:** Andrea J. Luker, Laura J. Graham, Timothy M. Smith, Carmen Camarena, Matt P. Zellner, Jamie-Jean S. Gilmer, Sheela R. Damle, Daniel H. Conrad, Harry D. Bear, Rebecca K. Martin

**Affiliations:** 1 Department of Microbiology and Immunology, School of Medicine, VCU, Box 980678, Richmond, VA 23298 USA; 2 Department of Biology, College of Humanities and Sciences, VCU, Richmond, VA USA; 3 Massey Cancer Center, VCU, Box 980678, Richmond, VA 23298 USA; 4 Division of Surgical Oncology, Department of Surgery, VCU, Richmond, VA USA

**Keywords:** Myeloid derived suppressor cells, DNA methyltransferase inhibitor, Immunotherapy, T lymphocytes, Myelopoiesis

## Abstract

**Background:**

Myeloid derived suppressor cells (MDSCs) present a significant obstacle to cancer immunotherapy because they dampen anti-tumor cytotoxic T cell responses. Previous groups, including our own, have reported on the myelo-depletive effects of certain chemotherapy agents. We have shown previously that decitabine increased tumor cell Class I and tumor antigen expression, increased ability of tumor cells to stimulate T lymphocytes, depleted tumor-induced MDSC in vivo and augmented immunotherapy of a murine mammary carcinoma.

**Results:**

In this study, we expand upon this observation by testing a next-generation DNA methyltransferase inhibitor (DNMTi), guadecitabine, which has increased stability in the circulation. Using the 4 T1 murine mammary carcinoma model, in BALB/cJ female mice, we found that guadecitabine significantly reduces tumor burden in a T cell-dependent manner by preventing excessive myeloid proliferation and systemic accumulation of MDSC. The remaining MDSC were shifted to an antigen-presenting phenotype. Building upon our previous publication, we show that guadecitabine enhances the therapeutic effect of adoptively transferred antigen-experienced lymphocytes to diminish tumor growth and improve overall survival. We also show guadecitabine’s versatility with similar tumor reduction and augmentation of immunotherapy in the C57BL/6 J E0771 murine breast cancer model.

**Conclusions:**

Guadecitabine depleted and altered MDSC, inhibited growth of two different murine mammary carcinomas in vivo*,* and augmented immunotherapeutic efficacy. Based on these findings, we believe the immune-modulatory effects of guadecitabine can help rescue anti-tumor immune response and contribute to the overall effectiveness of current cancer immunotherapies.

## Background

Tumors avoid immune detection and attack through a variety of mechanisms that circumvent the anti-tumor response. DNA hypermethylation, though reversible, can silence the expression of immunogenic antigens; this makes the immune system less effective, especially during immunotherapeutic interventions [1]. Tumors also recruit regulatory immune cells, including MDSCs, which dampen the adaptive immune response. Patients with higher levels of circulating MDSCs have increased primary tumor growth [2], higher metastatic burden [3], more advanced clinical cancer stage [4, 5], and shorter overall survival [3, 6]. Based on these findings and multiple reports in mouse models that implicate MDSCs as key obstacles to successful cancer immunotherapy, there has been much interest in eliminating the suppressive nature of MDSCs to improve patient outcomes [7–10].

The myeloid compartment in cancer has been extensively reviewed, especially MDSCs [11]. MDSCs are divided into two subsets, monocytic and granulocytic MDSCs. Both types of MDSCs have been shown to be suppressive in both murine tumor models and in several human cancers. Monocytic MDSCs generate nitric oxide as a mechanism of suppression, whereas granulocytic MDSCs express large amounts of reactive oxygen species and arginase-1 that results in suppression of T cell function [11]. MDSCs have been found in the spleen, blood, liver, and tumor of tumor-bearing animals. These suppressive cells have been found to accumulate in various types of murine tumor models and human cancer, from murine hepatic carcinoma models [12], breast cancer models [13], to human ovarian cancer [14], and many more.

Our lab has previously published on the effects of the DNA methyltransferase inhibitor (DNMTi), decitabine. Using the aggressive murine breast cancer line, 4 T1, we found that decitabine improved the immunogenicity of these cells in vitro, and augmented the effects of adoptive immunotherapy (AIT) in vivo [10]. While decitabine caused a reduction in tumor-induced MDSC accumulation, the underlying mechanism behind this was never investigated. In our current study, we have expanded upon these findings with the second-generation DNTMi, guadecitabine, and investigated its mechanism of action in tumor reduction. Like the active metabolite decitabine, we found that guadecitabine diminished tumor-induced granulocytosis in 4 T1 tumor-bearing mice. As a result of the reduced MDSC accumulation, guadecitabine rescued immune activation and was able to reduce tumor growth in a T cell-dependent manner. Guadecitabine was similarly effective in the E0771 model of murine breast carcinoma. Finally, we found that guadecitabine in combination with AIT resulted in prolonged survival in both 4 T1 and E0771 breast cancer models. Because of these advantageous effects, guadecitabine could prove to be a beneficial new drug to reduce systemic immune suppression and augment the effectiveness of immunotherapy in cancer patients.

## Results

### Guadecitabine treatment in vivo reduces tumor size and specifically targets the myeloid lineage with minimal effects on lymphoid populations

**Fig. 1:**
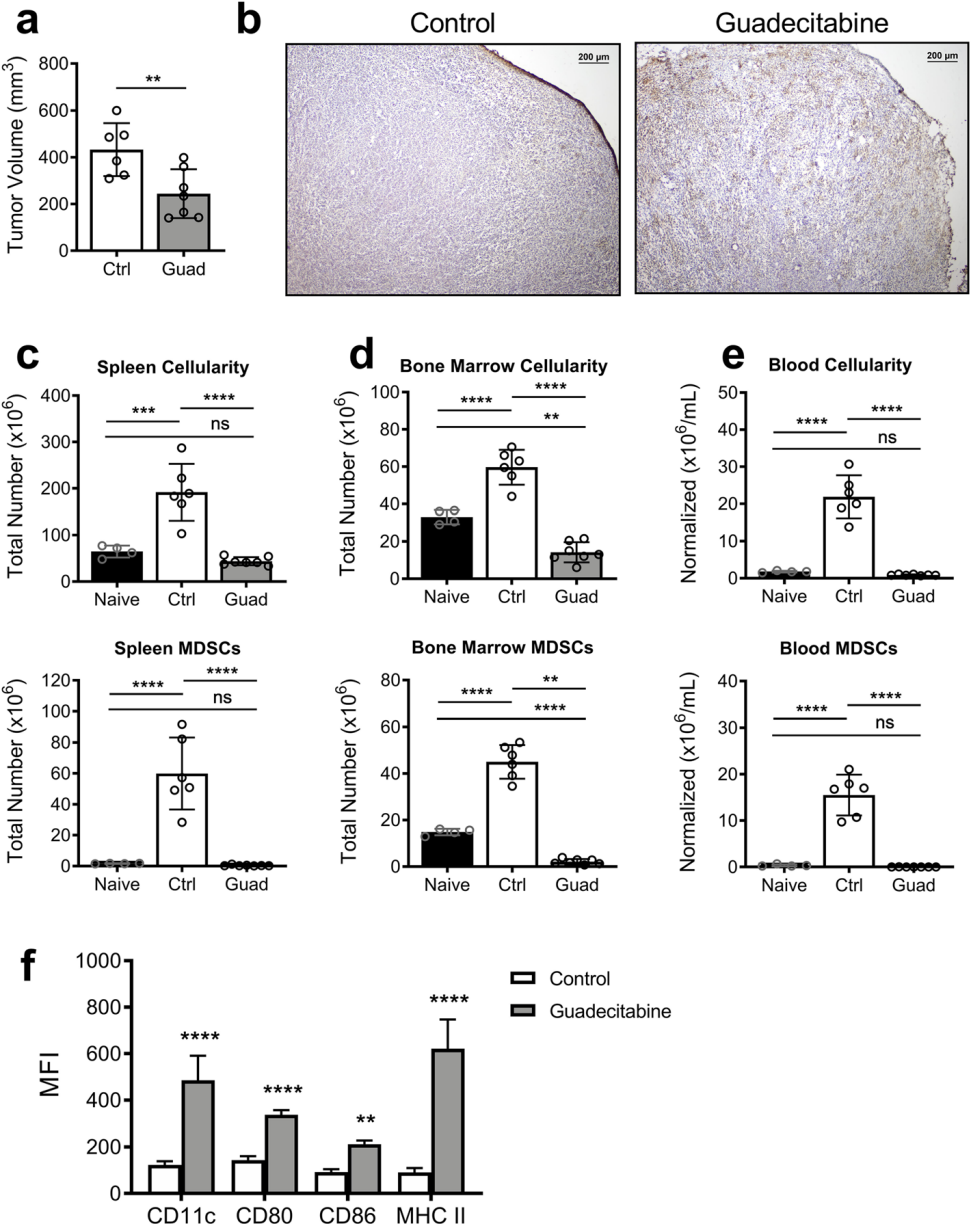
Guadecitabine treatment results in smaller tumors and a reduction in myeloid cells. 4 T1 tumor-bearing mice were untreated or treated with guadecitabine on days 10–13. **a** Final volume of excised tumors on day 16. **b** Representative images of frozen tumor sections stained with TUNEL to detect apoptotic cells. Total cellularity (top) and number of MDSCs (bottom) from **c** spleen, **d** bone marrow, and **e** blood. **f** Surface expression of APC costimulatory markers on splenic MDSCs. Significance determined using student’s unpaired T test (a), ANOVA with Tukey’s (c-e) or Sidak’s (f) multiple comparison tests. Error bars represent SD. ns = not significant; **:*p* value< 0.0021; ***:*p* value< 0.0002; ****:*p* value< 0.00001

4 T1 tumor-bearing WT Balb/cJ mice were treated daily on days 10, 11, 12, and 13 with 50 μg guadecitabine. By day 16, guadecitabine treatment had resulted in a significant reduction in tumor volume (Fig. 1a). Histologic examination revealed that control tumors had thick outer capsules surrounding the majority of the tumor, while tumors from guadecitabine-treated mice had thinner capsules that were often disrupted or fragmented. Tumors from guadecitabine-treated mice also had increased TUNEL^+^ apoptotic cells (Fig. 1b).

Progression of certain cancers can force the bone marrow and spleen into a phase of excessive myelopoiesis, whereby immature myeloid cells spill out into the circulation. This accumulation of myeloid cells is the underlying cause of the splenomegaly seen in the 4 T1 model, and provides a reservoir of recirculating MDSCs [7, 15, 16]. Indeed, within the spleens of control 4 T1 tumor-bearing mice we saw a large increase in cellularity due to a massive expansion of total MDSCs (Fig. 1c), with the granulocytic MDSCs accounting for 27.75% ± 1.627% of the total cell population (Supplemental Fig. 1a). There was a similar increase in total cellularity and number of MDSCs found in the bone marrow and blood (Fig. 1d,e), as has been previously reported [16–18]. Representative flow images for each treatment group and tissue ssample are shown in Supplemental Fig. 1. With guadecitabine treatment, however, the excessive myeloid populations were largely absent in each tissue compartment. In the remaining splenic MDSCs, we saw a significant increase in the immune-stimulatory markers MHC II, CD80, and CD86 (Fig. 1f). Together, these data suggest that guadecitabine depletes MDSCs by targeting excessive myelopoiesis. Additionally, guadecitabine appears to push remaining MDSCs toward a mature, antigen presenting cells (APC) phenotype.

To study the effects of guadecitabine on MDSCs further, we moved to an in vitro model. To do this we utilized MDSCs isolated from ADAM10Tg mice. These MDSCs result from a defect in hematopoiesis, do not overexpress ADAM10, are suppress T cells, express MDSC markers, are heterogeneous, have been extensively characterized, and are not tumor derived [19–22]. ADAM10Tg mice do not have any mature B cells. This makes them easy to harvest and isolate for experiments. MDSCs were treated in vitro with increasing doses of guadecitabine for 24, 48, or 72 h and then an MTT cytotoxicity assay was performed. Guadecitabine was only cytotoxic directly after extended periods of time (Supplemental Fig. 2a). The remaining MDSCs after 24 h of treatment had increased expression of MHC II, CD80, and CD86, similar to in vivo experiments, but only in the Ly6C^+^ population (Supplemental Fig. 2b). Next, we examined the direct effect on 4 T1 tumor cells in vitro, showing a direct cytotoxic effect only after a prolonged direct exposure to guadecitabine (Supplemental Fig. 2c). After 24 h of treatment, 4 T1 tumor cells showed increased MHC I expression most dramatically in the presence of IFNγ (Supplemental Fig. 2d). Using a T cell suppression assay, T cell proliferation was unaltered in the presence of MDSCs from ADAM10Tg mice treated with guadecitabine, as compared with MDSCs from vehicle treated mice that inhibited T cell proliferation (Supplemental Fig. 2e). This shows that MDSC survival and suppressive function are impaired by guadecitabine treatment.

While reducing suppressive myeloid cells can be beneficial, the lymphoid compartment is vital for anti-tumor immunity. Because of the robust MDSC expansion in the spleen, the percentage of B and T cells at day 16 was reduced in control tumor-bearing mice (Supplemental Fig. 3a,b,c). The absolute number of B and T cells was increased, suggesting immune activation. In guadecitabine-treated mice, the B and T cells were present at normal, although not elevated above, naïve levels. Additionally, the highly ordered structure of the spleen is essential to ensure proper cellular interactions. H&E staining illustrates that tumor-bearing control spleens have an enlargement of the red pulp due to accumulated MDSCs (Supplemental Fig. 3d). This expansion is absent with guadecitabine treatment, and importantly the spleens maintain appropriate separation of red and white pulp. Based on the cell numbers and intact architecture, guadecitabine does not appear to affect the splenic lymphoid populations. Due to the increase in BM immature myeloid cells that was reversed with guadecitabine treatment at day 16, the development of myeloid progenitors was examined in the BM. With guadecitabine treatment, the common myeloid progenitors (CMP) and megakaryocyte-erythrocyte progenitors (MEP) are significantly reduced, while the granulocyte-macrophage progenitors (GMP) are unaffected (Supplemental Fig. 3e).

**Fig. 2:**
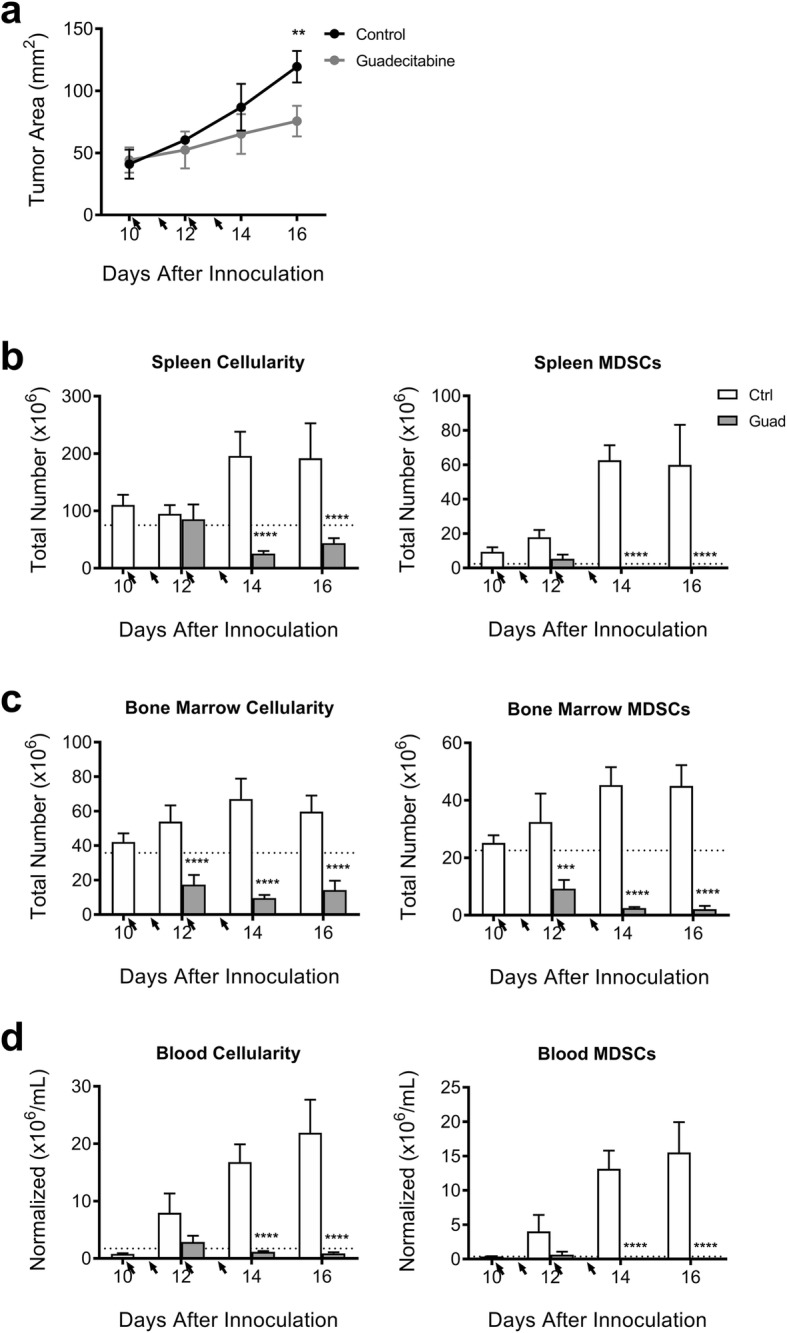
Guadecitabine slows tumor growth and immediately reverses the rapidly expanding myeloid population. **a** Timecourse experiment showing 4 T1 tumor growth in WT balb/c mice. Total cellularity and number of MDSCs from **b** spleen, **c** bone marrow, and **d** blood. Arrows indicate guadecitabine treatments; dotted line indicates naïve levels. Significance determined using ANOVA with Sidak’s multiple comparison tests. Error bars represent SD. ns = not significant, *:*p* value< 0.0332; **:*p* value< 0.0021; ***:*p* value< 0.0002; ****:*p* value< 0.00001

To investigate the temporal effects of guadecitabine, we next performed a time-course study. We observed an immediate slowing of tumor growth that reached significance by day 16 (Fig. 2a). Fig. 2b-d shows a steady increase in spleen, bone marrow, and blood cellularity in control tumor-bearing mice. MDSCs begin to accumulate in the bone marrow and blood around day 12, while the splenomegaly was slightly delayed until day 14. In each of these tissue compartments, however, guadecitabine instantly halted and reversed the accumulation of MDSCs. By day 16, the total MDSC populations were back to naïve levels.

### Guadecitabine’s effect on tumor growth is T cell-dependent

Cytotoxic T lymphocytes (CTLs) are the main effector cells responsible for cell-mediated killing of tumors. During an adaptive immune response, antigen-specific T cells become activated and expand to boost their anti-tumor activity. MDSCs have been shown many times to diminish the cytotoxic ability of CTLs in tumor-bearing hosts [7, 8, 23–29]. We therefore wanted to investigate whether the reduced tumor burden resulted from a direct effect of guadecitabine on the 4 T1 tumor cells in vivo or was secondary to the immunomodulatory effect of the drug.

**Fig. 3:**
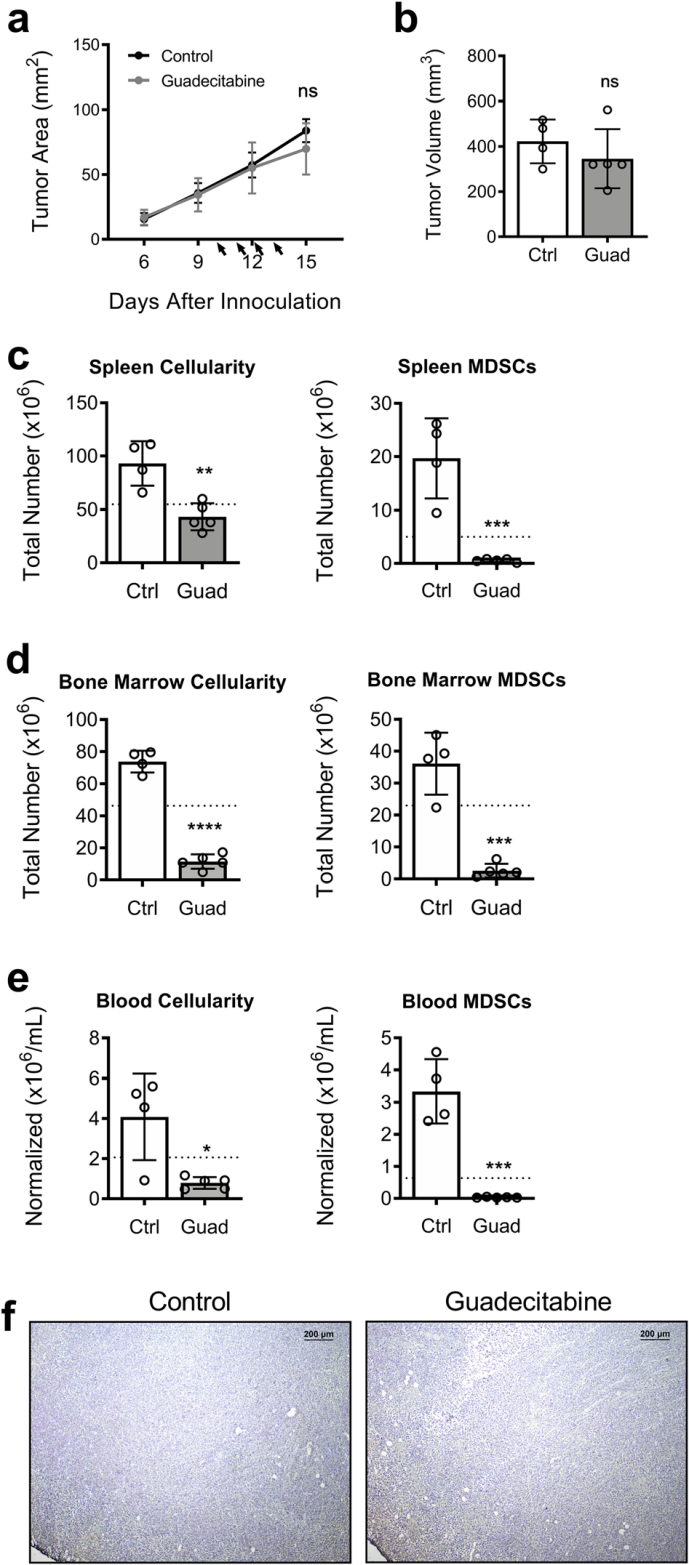
Tumor growth in T cell-deficient mice is not affected by guadecitabine. 4 T1 tumor challenge in athymic mice, showing **a** tumor growth curve and **b** final excised tumor volume at day 16. Total cellularity and number of MDSCs from **c** spleen, **d** bone marrow, and **e** blood. **f** Representative images of day 16 frozen tumor sections stained with TUNEL for apoptotic cells in T cell-deficient control mice or mice treated with guadecitabine, showing no clear difference in degree of apoptosis. Dotted line indicates naïve levels. Significance determined by unpaired student’s T-test. Error bars represent SD. ns = not significant; *:*p* value< 0.0332; **:*p* value< 0.0021; ***:*p* value< 0.0002; ****:*p* value< 0.00001

Athymic nude mice bearing 4 T1 tumors were either untreated or treated with guadecitabine as above. The tumors grew at an equal pace with or without guadecitabine (Fig. 3a,b). The treatments had the same effect of reversing the tumor-induced increase in cellularity and MDSC accumulation within the spleen, bone marrow, and circulation (Fig. 3c-e). TUNEL staining of the tumor sections, however, indicated few obvious apoptotic cells in either group (Fig. 3f), indicating that guadecitabine does not have a direct cytotoxic effect on 4 T1 tumors in vivo*.* Together, these data suggest that the effect of guadecitabine on tumor growth in vivo is T cell-dependent.

In order to confirm the role of T cells, we performed a series of depletion experiments to target and remove T cells with depletion antibodies according to the schedule in Supplemental Fig. 4a. We confirmed the specificity and completeness of the α-CD4 and α-CD8 depletions, showing that only the intended T cell populations were removed without affecting B cells and MDSCs (Supplemental Fig. 5a-c). As expected, mice receiving the isotype control + guadecitabine had smaller tumors than the isotype alone (Supplemental Fig. 4b). In mice which underwent CD8^+^ T cell depletion, however, we observed comparable tumor growth with or without guadecitabine treatment (Supplemental Fig. 4c). When CD4^+^, in addition to CD8^+^, cells were depleted, there was no additional effect on the tumor, suggesting CD4^+^ T cells do not play a significant role (Supplemental Fig. 4d). Together, these experiments confirm the role of T cells, but also indicate that CD8^+^ CTLs are the important population involved in the enhanced tumor immunity.

### Guadecitabine diminishes the T cell-inhibitory environment of the spleen

**Fig. 4:**
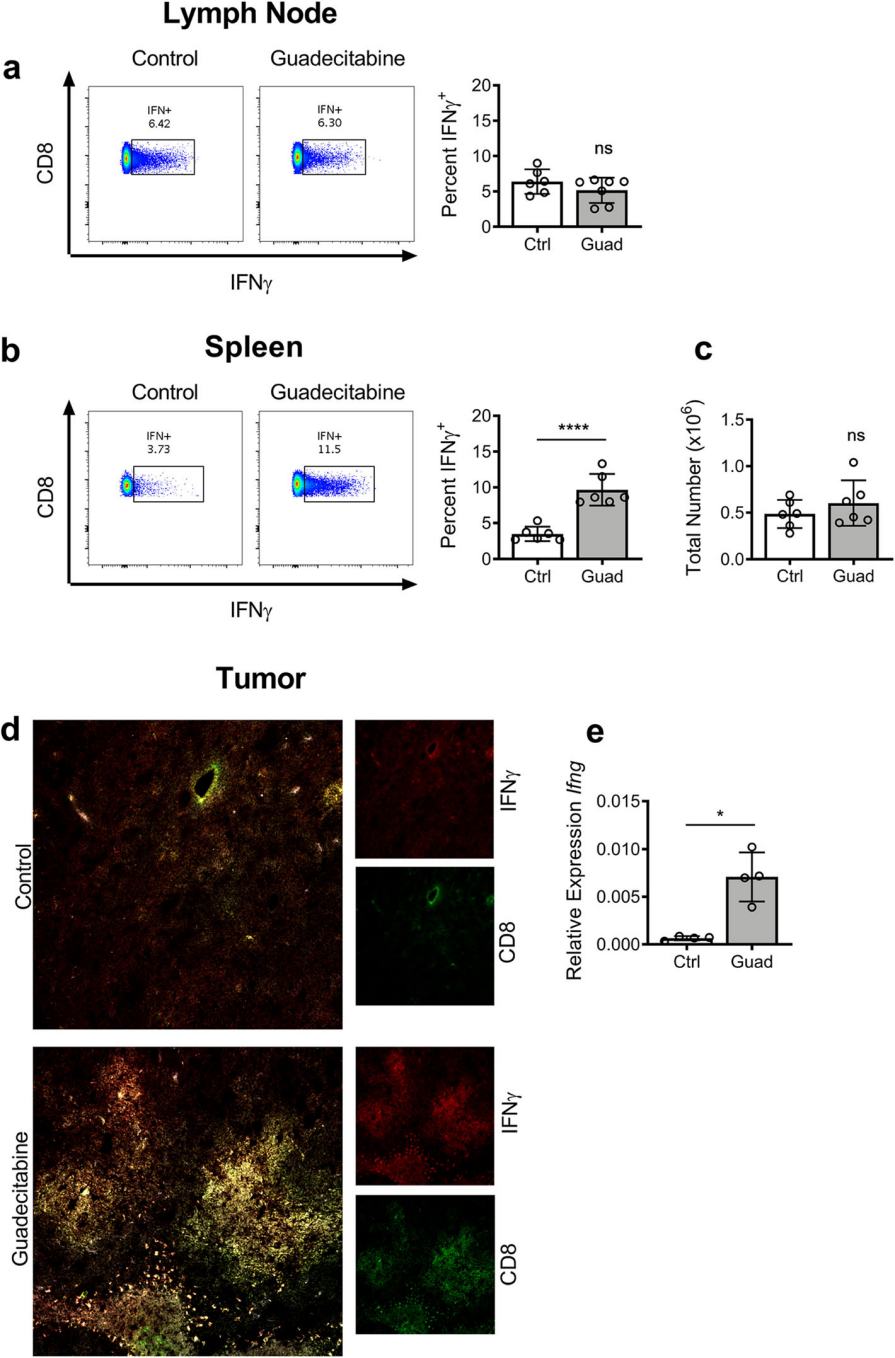
Guadecitabine boosts the CD8^+^ T cell anti-tumor response in the spleen. IFNγ production by **a** dLN lymphocytes and **b** splenocytes from tumor-bearing mice following ex vivo restimulation for 3 h with ionomycin and PMA. **c** Total number of IFNγ-producing CD8^+^ splenocytes. **d** Representative immunoflourescent staining of frozen tumor sections for anti-CD8α (green) and anti-IFNγ (blue). **e** Quantitative RT-PCR for *Ifng* message on whole tumor. Relative quatification normalized to housekeeping gene *Hrpt*. Significance determined by unpaired student’s T-test for a-c. Significance determined by Mann-Whitney non-parametric analysis for *E. error* bars represent SD. ns = not significant; *:*p* value< 0.0332; ****:*p* value< 0.00001

The draining lymph nodes (dLNs) are a site of robust immune activity and often of great interest in tumor studies [30]. We harvested dLN from WT tumor-bearing control or guadecitabine-treated mice on day 16 and restimulated the cells in vitro with ionomycin+PMA. Flow cytometry analysis showed no difference in the percent of CD8^+^ T cells producing IFNγ from guadecitabine-treated mice versus tumor-bearing controls (Fig. 4a). We did not observe MDSC infiltration into the dLN (Supplemental Fig. 6a) of these mice, leading us to conclude that T cells are being affected elsewhere.

Several groups have published on the importance of the spleen as a priming zone for T cell activity [7, 29]. Others have previously reported on the requirement of direct contact between MDSCs and T cells in order for suppression to occur [27, 29]. We therefore hypothesized that the MDSC accumulation within the spleen interacts with and suppresses CTLs as they recirculate. When day 16 splenocytes were restimulated in culture, there was a significant increase in the percent of CD8^+^ cells from guadecitabine-treated mice that produced IFNγ (Fig. 4b). To further investigate the T cell activity, we calculated the total number of IFN-producing CD8^+^ T cells between the groups and found no difference (Fig. 4c). This reveals that guadecitabine elicits a higher degree of activation from the same number of splenic CTLs. This enhanced activation is further evidenced by the greater proportion of IFNγ-producing cells within the spleen (9.663% ± 0.9034) compared to the highly active dLN (5.149% ± 0.6741). We also confirmed previous reports that MDSCs only affect CD8^+^ T cells [27], as we saw no effect on IFNγ production by CD4^+^ T cells (Supplemental Fig. 6b). As the tumor site is additionally an important interaction site for CTLs and MDSCs, we assessed frozen tumor sections for CD8a and IFNγ by immunoflourescent staining. Guadecitabine treatment increased both CD8a and IFNγ at the tumor site (Fig. 4d). This was confirmed with significantly elevated *Ifng* expression in guadecitabine treated tumor tissue (Fig. 4e). Next, we assessed MDSC activity and presence in the tumor and spleen and found that guadecitabine reduced arginase1 staining, and resulted in almost complete absence of Gr1 staining (Supplemental Fig. 7a,b).

### Used in combination with AIT, guadecitabine further slows tumor growth and prolongs overall survival

**Fig. 5:**
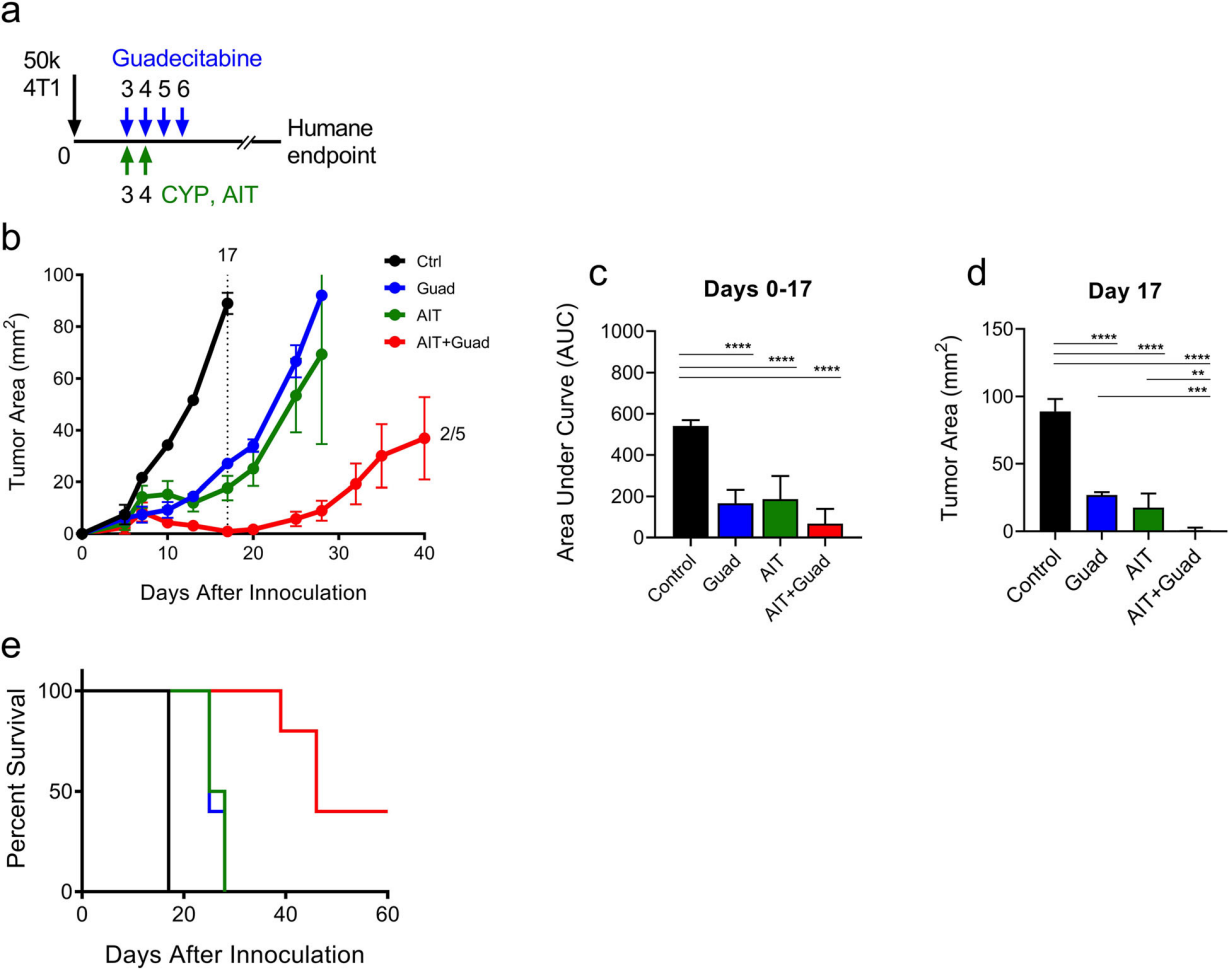
Therapy with guadecitabine and AIT slows tumor growth and improves overall survival compared to either treatment alone. **a** 4 T1 tumor-bearing mice were either untreated or treated with guadecitabine on days 3–6, and AIT mice then received CYP and 25 million antigen-experienced lymphocytes on days 3 and 4, respectively. **b** Tumor progression was measured until humane endpoints were reached; dotted line indicates day statistical significance was determined by **c** area under the curve or **d** tumor area. **e** Survival curves depicting overall survival in each treatment group. Significance determined using ANOVA with Tukey’s multiple comparison test Error bars represent SEM. *:*p* value< 0.0332; **:*p* value< 0.0021; ***:*p* value< 0.0002; ****:*p* value< 0.00001

We next tested the efficacy of guadecitabine administered in combination with the transfer of antigen-experienced lymphocytes. Lymphocytes from tumor-bearing donor mice were expanded ex vivo as previously described, resulting in 94.4% T cell purity (Supplemental Fig. 8a) [10]. Recipient animals were challenged with a 50,000-cell 4 T1 flank tumor on day 0 then treated as shown in Fig. 5a. Briefly, CYP and lymphocyte transfer coincided with the first treatments of guadecitabine on days 3 and 4. We observed a beneficial reduction in tumor size in mice that received guadecitabine or AIT alone. When combined, however, there was an impressive four-week delay in tumor growth, with complete regression in 2 of 5 mice (Fig. 5b) and improved survival (Fig. 5e). Statistical significance was determined up to day 17, when all treatment groups remained experimentally viable. By comparing the areas under the curves (AUC), each treatment group was significantly reduced compared to the control mice [31]. Additionally, the tumor measurements at day 17 show that the combination therapy resulted in significantly reduced tumor areas beyond guadecitabine or AIT alone. This separation of tumor growth curves continued to increase as the experiment progressed.

We also tested a different schedule, in which the CYP/AIT was delayed until the last treatment of guadecitabine on day 6 (Supplemental Fig. 8b); this allowed time for guadecitabine to take effect before the antigen-experienced lymphocytes were introduced and poses a greater challenge to the efficacy of AIT against larger tumors. In this case, the synergistic effect of guadecitabine and AIT persisted further out until day 40 (Supplemental Fig. 8c,d). In addition, 4 of 5 mice were cured, and we observed a higher overall survival when AIT occurred after guadecitabine (Supplemental Fig. 8f) with similar statistical significance (Supplemental Fig. 8d,e).

### Guadecitabine similarly reduces E0771 tumor burden

**Fig. 6:**
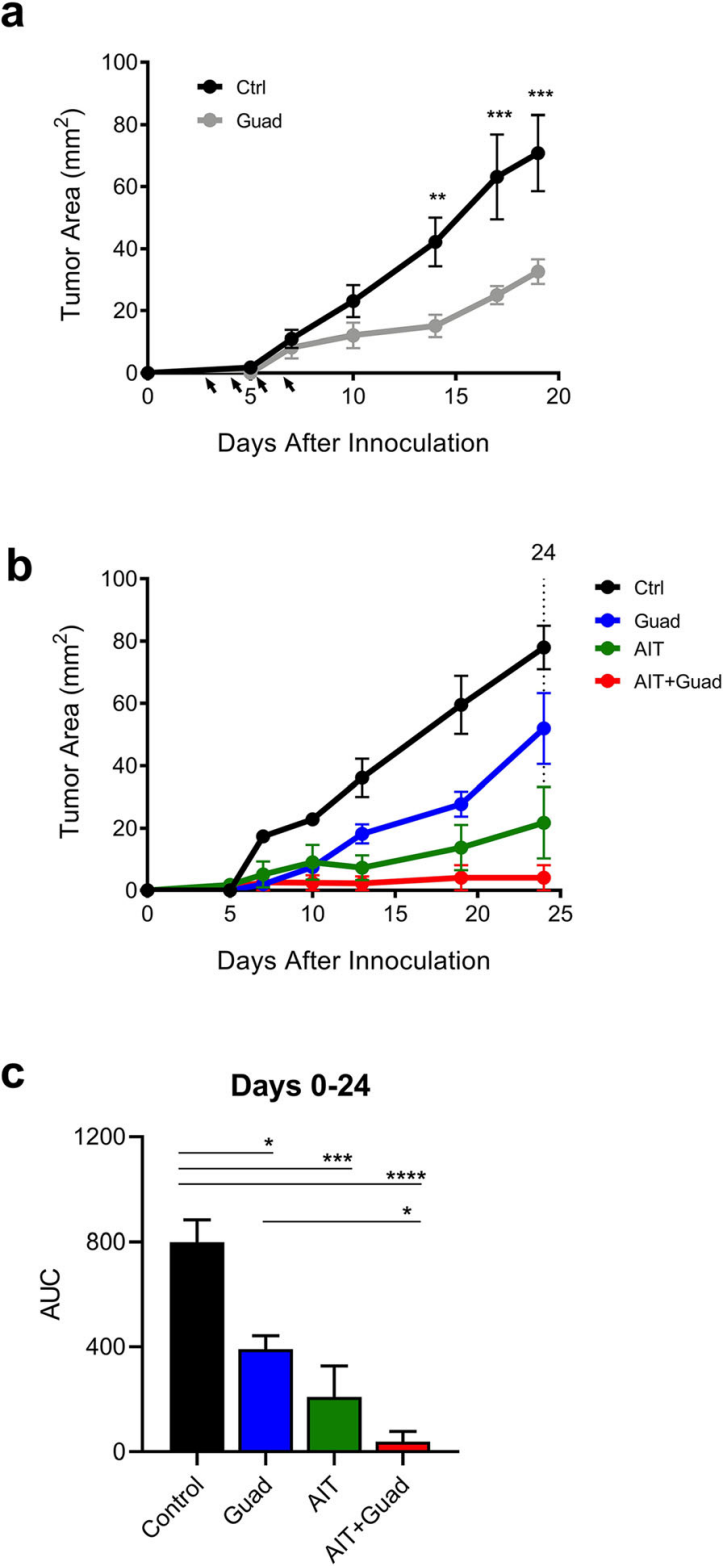
Guadecitabine similarly reduces E0771 tumor size and improves effectiveness of AIT. E0771 tumor-bearing mice were treated with guadecitabine on days 3–6. **a** Time-course experiment showing E0771 tumor growth in WT C57Bl/6 mice. **b** E0771 tumor progression following AIT treatment as in Fig. 5. **c** AUC quantification of tumor growth following combination therapy. Significance determined using ANOVA with Sidak’s (a) or Tukey’s (c) multiple comparison test. Error bars represent SEM. *:p value< 0.0332; **:*p* value< 0.0021; ***:*p* value< 0.0002; ****:*p* value< 0.00001

Finally, we wanted to test the effectiveness of guadecitabine in another breast cancer model. WT C57Bl/6 mice were injected subcutaneously with 200,000 E0771 cells. The tumors were allowed to become established for 3 days before the mice were treated daily on days 3, 4, 5, and 6 with 50 μg guadecitabine. Similar to the 4 T1 model, guadecitabine significantly reduced the growth of E0771 tumors (Fig. 6a). Additionally, adding guadecitabine significantly improved the impact of AIT (Fig. 6b,c).

## Discussion

There is great difficulty in treating neoplastic disease, especially when the tumor becomes resistant to chemotherapy. Today, the most promising interventions involve boosting the patient’s own immune system to detect and destroy abnormal cells. Immunotherapy has emerged as a highly promising and effective treatment for a variety of cancer types, but still only a minority of patients exhibit strong objective responses. Tumors can employ several “tactics” to avoid recognition and suppress anti-tumor immune response. Through hypermethylation, tumors can silence immunogenic antigens to avoid cell-mediated killing. Additionally, the tumor environment can induce massive myelopoiesis, causing suppressive MDSCs to accumulate in the bone marrow, spleen, circulation, and in the tumor. For these reasons, there is great interest in finding ways to reverse these effects, thus allowing the immune system to clear the tumor.

Several groups have reported on the myelo-depleting properties of demethylating drugs such as decitabine [28, 32] and 5-Azacitadine (AZA) [33], as well as other chemotherapy drugs, including gemcitabine [34, 35], doxorubicin [8] and docetaxel [36]. Although MDSCs are not being directly targeted, they seem to be more susceptible to their effects as demonstrated in this study. Guadecitabine, also known as SGI-110, was specifically designed to be resistant to degradation by cytidine deaminase and prolong the exposure of tumor cells to the active metabolite, decitabine. In vivo, guadecitabine treatments resulted in a near-complete absence of MDSCs in tumor-bearing mice (Fig. 1c-e). Based on the time-course experiment (Fig. 2), it appears that guadecitabine treatment is *preventing*, rather than reversing, MDSC accumulation. We believe guadecitabine targets the bone marrow by diminishing the highly proliferative myeloid progenitors (Fig. 2c, Supplemental Fig. 3e). This prevents increased MDSC circulation (Fig. 2d) and accumulation within the spleen (Fig. 2b). Surprisingly, we found that the similarly proliferative 4 T1 tumor cells were not vulnerable to cytotoxic effects of in vivo guadecitabine treatments (Fig. 3f).

Within the spleen of tumor-bearing mice we showed an accumulation of MDSCs in the red pulp (Supplemental Fig. 2d). This perilymphoid localization puts the MDSCs in contact with recirculating CD8^+^ CTL. Several tumor studies have portrayed the spleen as an inhibitory environment that can diminish CTL function [7, 37]. In experiments by Ugel et al, the investigators removed the inhibitory MDSC environment through splenectomies [7]. Although this did not affect tumor size, they found that T cell activation was recovered despite normal MDSC frequency within the blood and other tissues. This highlights the spleen’s unique role as an isolated region of suppression with the ability to severely dampen the anti-tumor immune response. In the present study, we used guadecitabine to ablate the suppressive splenic environment. We found that IFNγ production within the dLN is comparable between control and guadecitabine-treated mice (Fig. 4a). Upon recirculating through the spleen, however, CTLs from control tumor-bearing mice have diminished activation (Fig. 4b), even though the number of activated cells remained the same (Fig. 4c). These data support the role of the spleen as an important suppressive zone that contributes to tumor progression.

Unlike Ugel’s splenectomy experiments, our treatment additionally resulted in slower tumor growth (Fig. 2a), indicating guadecitabine may have a beneficial impact beyond the removal of regulatory myeloid populations from the spleen. The reduced suppressive activity in the spleen and tumor environments may be the reason for the reduction in tumor growth (Supplementary Fig. 7a,b). A recent study examining MDSC subsets in the peripheral blood of patients with multiple types of cancer found that arginase1 generated by granulocytic MDSCs was the main T cell inhibition mechanism [38]. In our study, guadecitabine was able to dramatically reverse the generation of arginase1, as well as dramatically reducing granulocytic MDSCs. Additionally, we demonstrated increased *Ifng* expression in the tumors of guadecitabine-treated mice (Fig. 4e). Kim et al. recently demonstrated across several murine models of triple negative breast cancer (TNBC) and in patient samples of TNBC that tumor infiltrating neutrophilic myeloid cells (TINs) were immunosuppressive and contribute to poor prognostic outcomes in patients [13]. In contrast, tumor infiltrating macrophages or monocytic myeloid cells (TIMs) were associated with increased responsiveness to checkpoint inhibitors in the murine models and better prognosis in humans [13]. Using guadecitabine treatment, Gr1^+^ cells are depleted, which include both MDSCs and neutrophils, but beneficial F4/80^+^ TIMs are not affected (Supplemental Fig. 7b). The enhanced tumor immunity may additionally arise from guadecitabine’s effect on MDSC phenotype. Although the majority of the MDSCs are eliminated, a small percentage of cells remained that are induced to express APC and costimulatory markers such as MHC II and CD80/86 (Fig. 1f, Supplemental Fig. 1b). Since myeloid populations have been previously shown to be extremely plastic [39], these data suggest that guadecitabine pushes suppressive MDSCs to develop into an immune-stimulatory phenotype that may augment immune activation within the spleen.

The Ugel experiment also emphasizes a significant problem with a popular and promising clinical therapy. Animals that underwent sham surgeries responded poorly to AIT compared to those that received splenectomies. When the antigen-experienced T cells circulate through the suppressive splenin environment, they are inactivated despite being primed to target the tumor. Here we have shown a similar phenomenon; while AIT was effective in slowing the growth rate of tumors, combination therapy with AIT+guadecitabine compounded this effect and resulted in persistent tumor suppression (Fig. 5b) and prolonged survival (Fig. 5e). It is interesting to note that in the AIT experiments, guadecitabine was administered earlier at days 3, 4, 5, and 6 (Fig. 5), rather than days 10, 11, 12, and 13 (Figs. 1, 2, 3 and 4). This dosing schedule still resulted in slower tumor growth through day 16, although the reasons why are unclear. Further, we tested the effectiveness of delaying adoptive T cell transfer until the final guadecitabine treatment rather than being delivered concomitantly with the initial treatment (Supplemental Fig. 7). The delayed AIT alone was more effective at days 6 and 7 than at days 3 and 4, perhaps because the tumor has become more immunogenic and vascularized by the later date.

Chimeric antigen receptor (CAR) T cells are genetically engineered to be specific for a designated target tumor antigen. These cells have been shown to be successful in the clinic against CD19^+^ B cell neoplasms. New CARs are being generated against antigens on solid tumors [40]. One of the biggest blocks to CAR treatment in solid tumors has been the suppressive environment created by MDSCs [40]. The addition of guadecitabine to CAR therapy may hold the key to favorably altering this environment and, as seen with the AIT experiments in this study, help augment the efficacy of adoptively transferred T cells.

Finally, we showed the efficacy of guadecitabine in slowing the growth of another tumor line on a different background strain. There were no observable strain differences noted relating to guadecitabine treatment between the C57Bl/6 J and Balb/cJ mice. The tumor line E0771 is not known to elicit a robust leukemoid reaction, but studies still indicate a suppressive role for MDSCs in this model [41, 42]. Overall, we observed a similar and persistent reduction in tumor growth with guadecitabine alone, or in combination with AIT.

## Conclusions

In conclusion, we have shown that guadecitabine treatment is effective against the aggressive 4 T1 and E0771 breast cancer lines targets excessive myelopoiesis within the bone marrow and greatly reduces MDSCs within the spleen, tumor, and blood. This eliminates the systemic T cell suppression, thereby rescuing the host’s anti-tumor CTL response. We’ve shown that guadecitabine treatment significantly improves AIT treatment by providing an environment in which the transferred T cells can maintain their antigen-specific activity long-term. Because of these advantageous effects of guadecitabine on multiple targets, it should prove to be a beneficial new drug to reduce systemic immune suppression and augment the effectiveness of immunotherapy in cancer patients. The immunosuppression relief provided by guadecitabine may also enhance responsiveness to other cancer treatments such as checkpoint inhibitors or CAR T cells that are inhibited by suppressive myeloid cells.

## Methods

### Animals

Wildtype (WT) female Balb/cJ, C57Bl/6 J, and athymic NU/J mice 8–10 weeks old were purchased from Jackson Laboratory. The health report was consistent with that of our Barrier Vivarium facility. Balb/cJ mice weighed an average of 20 g prior to the start of experiments and C57Bl/6 J mice weighed an average of 20 g prior to the start of experiments. ADAM10Tg mice were generated by the VCU Transgenic Core and maintained in the Barrier Vivarium facility. All mice were housed within Virginia Commonwealth University vivarium facilities, specifically the Massey Cancer Center Barrier Vivarium, in accordance with the humane treatment of laboratory animals set forth by the NIH and the American Association for the Accreditation of Laboratory Animal Care (AAALAC). All animal experiments were conducted with the permission and oversight of the Virginia Commonwealth University Institutional Animal Care and Use Committee (IACUC) under the protocols AM10065, and AM10256.

All animals were housed with 12 h light and dark cycle in NexGen Cages from Allentown (194 mm × 181 mm × 398 mm) on ventilated racks with corncob bedding (Shepard’s Specialty Corn Cob Plus). The temperature maintained in the cages is between 68 to 76 degrees Fahrenheit. Five animals are housed per cage. Animals are fed Envigo Teklad 2919 and given water ad libitum via Lab Product’s Hydropacs.

Animals were euthanized by CO_2_ inhalation followed by cervical dislocation.

### Experimental models and guadecitabine treatment

50,000 4 T1 or 200,000 E0771 cells in 50 μL PBS were injected subcutaneously into the flank at day 0. Cagemates were randomly assigned to differing groups at the start of the experiment. Appropriate groups received *i.p.* injections of 50 μg guadecitabine (kindly provided by Astex Pharmaceuticals, Inc.) on days 10, 11, 12, and 13, unless otherwise indicated. All injections took place between the hours of 10 am to 2 pm, working from the home cage. For each treatment, the control group was initially treated, followed by the non-control groups. Mice were euthanized on day 16, when we collected blood by cardiac puncture, bone marrow from femurs and tibias, tumors, spleens, and inguinal lymph nodes.

T cell depletion was performed as previously described [20]. Briefly, mice were injected *i.p.* with 200 μg of monoclonal antibodies on days 6, 7, 8, 9, and 14. Upon sacrifice, T cell depletion was confirmed in the spleen by flow cytometry. CD4^+^ and CD8^+^ T cells were depleted using the clones GK1.5 and 2.43, respectively (antibodies generated in house). Rat IgG was used as an isotype control.

AIT was performed as previously described [10]. Briefly, donor Balb/cJ or C57Bl/6 mice were injected with 5 × 10^5^ 4 T1 or E0771 cells, respectively, into the hind footpad; popliteal lymph nodes were collected at day 10 and activated overnight with bryostatin (5 nM, Calbiochem) and ionomycin (1 μM, Calbiochem) in the presence of recombinant IL-2 (Peprotech). Cells were then washed and expanded in IL-7 and IL-15 (both 10 ng/mL, Peprotech) for one week. On the indicated day, tumor-bearing recipient mice were treated *i.p.* with 100 mg/kg of cyclophosphamide (CYP). 24 h later, 50 million expanded lymphocytes were infused intravenously. All groups, except control mice, received a single CYP treatment. Tumor areas were measured through the skin on live animals using digital calipers as length x width; tumor volumes represent length x width x height of excised tumors. Animals were observed at least three times per week, according to IACUC standards. Animals were euthanized upon reaching a humane endpoint, including tumor area > 100 mm^2^, severe ulceration, or weight-loss.

ADAM10Tg animals and C57Bl/6 animals used for MDSC and T cell isolation were euthanized and spleens were harvested.

### Organ processing and cell counts

Blood volume collected by cardiac puncture was recorded and used to calculate the normalized number of cells per milliliter. Whole spleens were crushed to obtain a single cell suspension, then red blood cells were removed with ACK lysing buffer (Quality Biological). Femurs and tibias from each mouse were cleaned of connective tissue and spun at 350×g for 5 min to collect marrow before removing red blood cells. Viable cell counts were performed using trypan blue exclusions.

### Magnetic cell isolation, T cell suppression assay, and MMT cytotoxicity assay

Splenic MDSCs were purified by CD90.2 and CD11c magnetic depletion (Miltenyi Biotec). In experiments where MDSCs are considered pretreated, ADAM10Tg mice received four consecutive daily i.p. injections of vehicle or guadecitabine before collecting splenic MDSCs. CD90.2^+^ T cells were purified by magnetic column (Miltenyi Biotec) then labeled with Track-It Violet (Biolegend). Cells were co-cultured with MDSCs from control or guadecitabine-treated ADAM10Tg mice at a 1:2 (T/MDSC) ratio in the presence of anti-CD28 and plate-bound anti-CD3 (both 1 μg/mL, Biolegend) for 96 h. Cells were then harvested and analyzed by flow cytometry for T cell division.

The MTT assay was performed according to manufacturer’s instructions (Abcam). Both 4 T1 cells and MDSCs were cultured with increasing doses of guadecitabine for either 24, 48, or 72 h prior to performing the assay.

### Flow cytometry

Single cell suspensions were obtained and stained with the fixable live/dead stain ZombieAqua (Biolegend) per manufacturer’s instructions. Samples were then Fc-blocked with 2.4G2 [43] for 5 min and stained for 30 min on ice. Flow samples that included multiple Brilliant Violet antibodies were stained in the presence of Brilliant Stain Buffer (BD Biosciences) per manufacturer’s instructions. All cells were then fixed in 4% paraformalydehyde (PFA) fixation buffer (Biolegend) for 15 min at room temp. For intracellular staining, fixed cells were permeabilized with PermWash Buffer (Biolegend) per manufacturer’s instructions. Flow data were collected using a BD LSRFortessa running BDFACSDiva™ 8.0 software, and analyzed with FlowJo (10.4.2). Total MDSCs were characterized as both monocytic and granulocytic populations combined. Gating for MDSC populations are as follows: CD11b^+^ Ly6C^hi^ (monocytic-MDSCs) and CD11b^+^ Ly6C^int^Ly6G^+^ (granulocytic MDSCs). B cells were gated as MHC II^+^ B220^+^, and T cells were gated as B220^−^ CD4^+^ or CD8^+^. CMP, GMP, and MEP were gated as previously described [44]. The antibody clones were as follows: Ly6C (clone HK1.4), Ly6G (1A8), IFNγ (XMG1.2), CD80 (16-10A1), CD86 (GL-1), I-A/I-E (M5/114.15.2), Sca-1 (D7), CD16/32 (2.4G2), cKit (2B8) Lineage cocktail (B220 (RA3-6B2), CD4 (GK1.5), CD8a (53–6.7), Gr1 (RB6-8C5), CD11b (M1/70), Ter119 (Ter119)), IL7Rα (A7R34), CD34 (MEC14.7) all from Biolegend, and CD45R/B220 (RA3-6B2), CD4 (GK1.5), CD11b (M1/70), all from BD Biosciences.

### Histology and Immunoflourescence

Some spleens and tumors were fixed with 4% PFA for 15 min then equilibrated with successive incubations in 10, 20, and 30% sucrose before being mounted in Optimal Cutting Temperature (OCT) medium. 10 μm cryo-sections were briefly fixed in ice-cold acetone then in 4% PFA prior to staining. Light microscopy slides were stained using ApopTag Plus Peroxidase In Situ Apoptosis Detection Kit (Millipore Sigma) followed by counterstaining with Meyer’s Hematoxylin then imaged using an Olympus BX41. Immunoflourescence slides were stained as previously described [45] with antibody clones arginase1 (clone C-2–Santa Cruz Biotechnology), F4/80 (BM8), IFNγ (XMG1.2), Gr1 (RB6-8C5), and CD8a (53–6.7) all from Biolegend. They were imaged on a Zeiss LSM 700 confocal microscope and images were processed on Zeiss 3.1 Blue edition software.

### qRT-PCR

For RNA isolation from tumors, TRIzol (Invitrogen) was added to frozen tissue and homogenized. Subsequent RNA isolation was performed according to the manufacturer’s instructions. RNA was quantified using an ND-100 NanoDrop spectrophotometer. One microgram of total RNA was reverse transcribed using SuperScript IV (Thermo Fisher) with oligo (dT20). Primers used in quantitative PCR (qPCR) analysis are as follows: *Hprt*_forward 5′-CAGGGATTTGAATCACGTTTGTG-3′, *Hprt*_reverse 5′-TTGCAGATTCAACTTGCGCT-3′, *Ifng*_forward 5′-TGCCAAGTTTGAGGTCAACAAC-3′, *Ifng*_reverse 5′-TCATTGAATGCTTGGCGCTG-3′, In short, qPCR was conducted using a QuantStudio 3 Real-Time PCR System with 45 cycles using PowerUp SYBR Green Master Mix (both from Applied Biosystems). Primers were tested for specificity using melt curve analysis.

### Cell lines

4 T1 (ATCC® CRL-259™) and E0771 cell lines were purchased from ATCC and CH3 Biosystems, respectively. Cell lines were maintained at low passage numbers and ATCC-recommended tests were performed, including morphology checks and mycoplasma screening.

### Ex vivo restimulation for IFNγ production

All cells from each individual tumor-draining lymph node (dLN) or 10^6^ total splenocytes were plated in 2 mL media and restimulated with PMA (250 ng/mL) and ionomycin (1 μM) in the presence of monensin and brefeldin A (Biolegend). After three hours, the cells were washed and permeabilized before being stained for intracellular IFNγ for flow cytometry.

### Statistics

Each figure depicts one representative experiment of at least three independently conducted experiments. *n* values vary between 3 and 5 animals per group, per experiment. The number of experimental animals was determined based on data from spread in previous tumor experiments, while taking into account the need to reduce the use of unnecessary animals in research. For un-paired comparisons, a Student’s t test was performed. Where appropriate, a one-way ANOVA with Tukey’s or a two-way ANOVA with Sidak’s multiple comparison tests was used when analyzing 3+ normally distributed data-sets (specific test indicated in legend). All statistical analyses were performed using GraphPad Prism 7. Significance is indicated in individual figure legends.

## Supplementary information

**Additional file 1: Supplemental Figure 1.** MDSC final gating for flow cytometry, comparing different sites from control and guadecitabine-treated 4T1 tumor-bearing mice and naïve mice. All samples were run as single-cell suspensions, followed by doublet- and dead cell-exclusion. Gated on CD11b^+^ live singlets, CD11b^+^ cells were then visualized as Ly6C^hi^ or Ly6C^int^ Ly6G^+^ populations. Percentage and total number of MDSCs was calculated by combining both of these populations. Data is from one experiment and is representative. **Supplemental Figure 2.** Guadecitabine alters expression and function of MDSCs *in vitro*. a. MDSC cytotoxicity with increasing doses of guadecitabine over 24hours (triangles), 48hours (diamond), and 72 hours (circles) of treatment. b. Guadecitabine treatment and upregulation of surface expression of MHCII, CD80, and CD80 on MDSCs (Ly6C+; open bars, Ly6G+; closed bars) *in vitro* by flow cytometry. c. 4T1 cytotoxicity with increasing doses of guadecitabine over 24hours (triangles), 48hours (diamond), and 72 hours (circles) of treatment. d. MHCI expression on 4TI cells following guadecitabine treatment for 24 hours ± 5ng/mL IFNγ treatment. e. MDSC activity as measured by T cell suppression after *in vivo* pre-treatment of ADAM10Tg mice with guadecitabine or vehicle. MDSC experiments are n=3 at each data point. 4T1 experiments are n=4 at each data point. Statistical tests were performed by Two-Way ANOVA (a, c). One-Way ANOVA (b, d). For a. and c. top stars apply to comparison between 72 and 48 hours, lower stars apply to comparison between 72 and 24 hours, and the bottom stars apply to comparison between 24 and 48 hours at that particular dosage. e. Representative of n=3. *:p<0.0332; **:p<0.0021 ; ***:p<0.0002; ****:p<0.0001. **Supplemental Figure 3.** Analysis of cellular populations of the spleen and bone marrow of tumor-bearing mice with or without guadecitabine treatment. a. Differential cell analysis showing percentage (left) and total number (right) of splenocyte populations. Percent and total number of B cells (b.) and T cells (c.) within the spleen. d. Representative image of frozen spleen sections stained with H&E. e. Percent of bone marrow (BM) progenitors from tumor bearing animals treated with guadecitabine or control were determined by flow cytometery. All were gated on lineage^-^, live, singlets. Common myeloid progenitors (CMP) (IL7Rα^-^,cKit^+^,Sca1^-^,CD16/32^-^,CD34^+^), granulocyte-macrophage progenitors (GMP) (IL7Rα^-^,cKit^+^,Sca1^-^,CD16/32^+^,CD34^+^), and megakaryocyte-erythrocyte progenitors (MEP) (IL7Rα^-^,cKit^+^,Sca1^-^,CD16/32^-^,CD34^-^) were determined. Representative of three experimental replicates, n=4 for naïve, n=6 for control, and n=6 for guadecitabine, (a-d). n=3-5/group for (e). Significance was determined using an ANOVA with a Tukey’s multiple comparison (for a-c) or a student’s T test (e). Error bars represent SD **:p value<0.0021; ****:p value<0.00001; ns:not significant. **Supplemental Figure 4.** Confirming the role of T cells. a. T cell depletion treatment schedule. Tumor growth and final volume in mice treated with rat IgG isotype control (b), anti-CD8 depletion antibody (c), and anti-CD4/anti-CD8 depletion antibodies (d). Representative results from two independent experiments. Statistical significance determined by unpaired student’s T-test. Error bars represent SD. ns: not significant; *:p value<0.0332. **Supplemental Figure 5.** Confirmation of T cell depletion. Representative flow cytometry analysis of day 16 splenocytes following treatment with rat IgG isotype control (a), anti-CD8 depletion antibody (b), and anti-CD4/anti-CD8 depletion antibodies (c). **Supplemental Figure 6.** Differential dLN populations and CD4 ex vivo restimulation. a. Differential cell analysis showing percentage (left) and total number (right) of dLN populations. b. IFNγ production by CD4^+^ cells from dLN (left) or spleen (right) following ex vivo restimulation with PMA and ionomycin. n=3 mice/group for (a). Representative flow cytometry populations in (b). **Supplemental Figure 7.** MDSC activity as measured by Arginase1 staining in the spleen and tumor. Spleens (a) and tumors (b) from D16 were sectioned and stained for Arginase1 (blue), Gr1 (red), and F4/80 (green). Representative images of 4 slides per group. . **Supplemental Figure 8.** AIT alternative dosing schedule in 4T1 tumor model. a. T cell purity from 7 day expansion of lymphocytes from dLN of tumor-bearing donor mice. Representative image from one AIT experiment. Gated on live, singlets. b. 4T1-tumor bearing mice were treated with guadecitabine on days 3-6, then received CYP and 25 million antigen-experienced lymphocytes on days 6,7. c. Tumor progression was measured until humane endpoints were researched; dotted line indicates day statistical significance was determined by area under the curve (d) or tumor area (e). f. Survival curves depict overall survival in each treatment group. n>5 mice/group. Significance determined using ANOVA with Tukey’s multiple comparison test. Error bars represent SEM. *:p value<0.0332; ***:p value<0.0002; ****:p value<0.00001.

## Data Availability

The datasets used and/or analyzed during the current study available from the corresponding author on reasonable request.

## Abbreviations

AIT: Adoptive immunotherapy
APC: Antigen presenting cells
AUC: Area under the curve
AZA: 5-Azacitadine
BM: Bone Marrow
CTL: Cytotoxic T lymphocytes
CYP: Cyclophosphamide
dLN: Draining lymph node
DNMTi: DNA methyltransferase inhibitor
OCT: Optimal cutting temperature
PFA: Paraformaldehyde
TNBC: Triple negative breast cancer
WT: Wildtype
