# Supplementary material for: The DNA methyltransferase inhibitor, guadecitabine, targets tumor-induced myelopoiesis and recovers T cell activity to slow tumor growth in combination with adoptive immunotherapy in a mouse model of breast cancer
Source: BMC Immunol. 2020 Feb 27;21:8. doi: 10.1186/s12865-020-0337-5 (PMC7045411; doi:10.1186/s12865-020-0337-5)
Supplement: Supplementary file 1 — Additional file 1: Supplemental Figure 1. MDSC final gating for flow cytometry, comparing different sites from control and guadecitabine-treated 4T1 tumor-bearing mice and naïve mice. All samples were run as single-cell suspensions, followed by doublet- and dead cell-exclusion. Gated on CD11b+ live singlets, CD11b+ cells were then visualized as Ly6Chi or Ly6Cint Ly6G+ populations. Percentage and total number of MDSCs was calculated by combining both of these populations. Data is from one experiment and is representative. Supplemental Figure 2. Guadecitabine alters expression and function of MDSCs in vitro. a. MDSC cytotoxicity with increasing doses of guadecitabine over 24hours (triangles), 48hours (diamond), and 72 hours (circles) of treatment. b. Guadecitabine treatment and upregulation of surface expression of MHCII, CD80, and CD80 on MDSCs (Ly6C+; open bars, Ly6G+; closed bars) in vitro by flow cytometry. c. 4T1 cytotoxicity with increasing doses of guadecitabine over 24hours (triangles), 48hours (diamond), and 72 hours (circles) of treatment. d. MHCI expression on 4TI cells following guadecitabine treatment for 24 hours ± 5ng/mL IFNγ treatment. e. MDSC activity as measured by T cell suppression after in vivo pre-treatment of ADAM10Tg mice with guadecitabine or vehicle. MDSC experiments are n=3 at each data point. 4T1 experiments are n=4 at each data point. Statistical tests were performed by Two-Way ANOVA (a, c). One-Way ANOVA (b, d). For a. and c. top stars apply to comparison between 72 and 48 hours, lower stars apply to comparison between 72 and 24 hours, and the bottom stars apply to comparison between 24 and 48 hours at that particular dosage. e. Representative of n=3. *:p<0.0332; **:p<0.0021 ; ***:p<0.0002; ****:p<0.0001. Supplemental Figure 3. Analysis of cellular populations of the spleen and bone marrow of tumor-bearing mice with or without guadecitabine treatment. a. Differential cell analysis showing percentage (left) and total number (right) of sple [file 12865_2020_337_MOESM1_ESM.docx]

Supplemental Figure 1


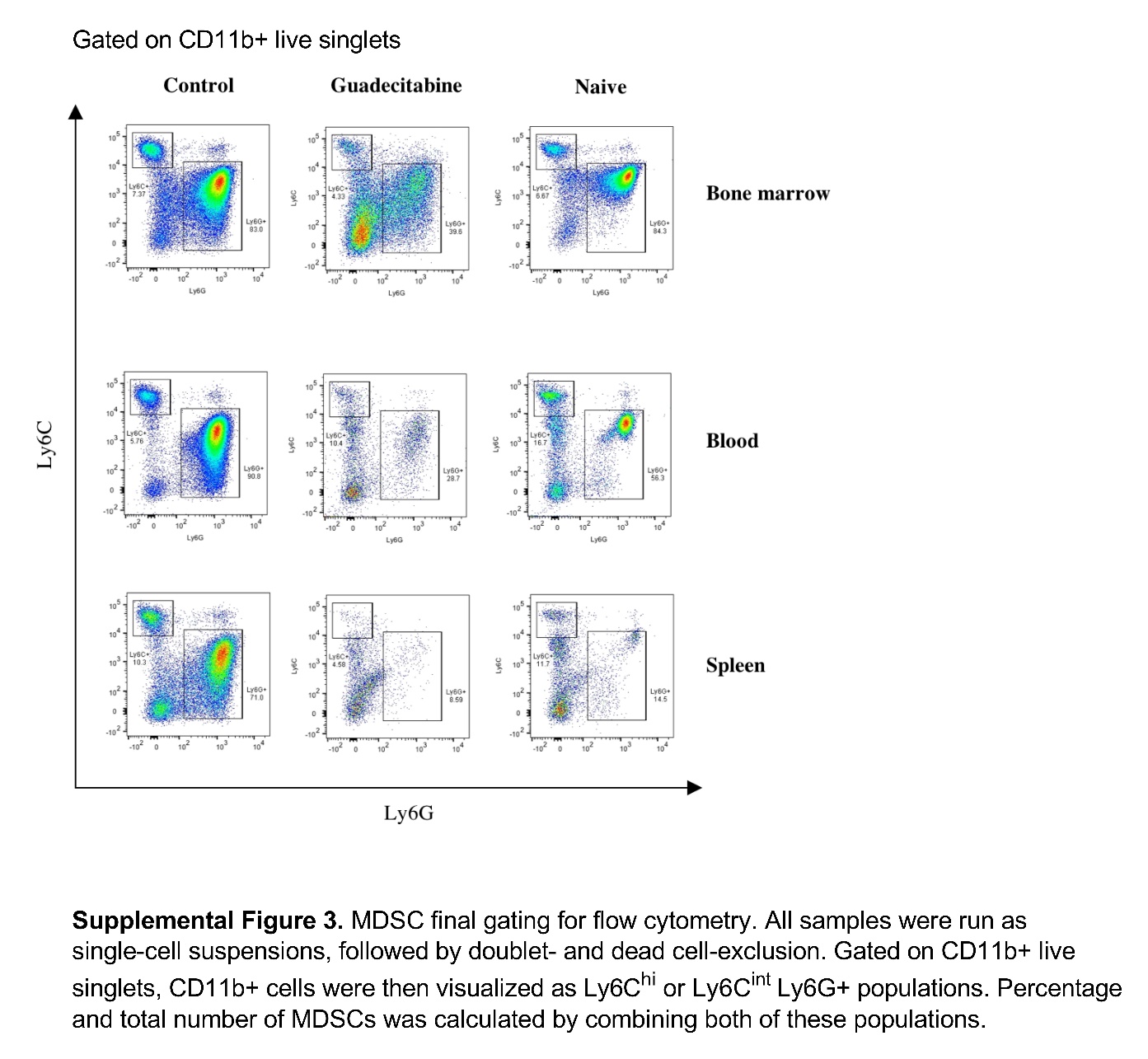


**Supplemental Figure 1.** MDSC final gating for flow cytometry, comparing different sites from control and guadecitabine-treated 4T1 tumor-bearing mice and naïve mice. All samples were run as single-cell suspensions, followed by doublet- and dead cell-exclusion. Gated on CD11b^+^ live singlets, CD11b^+^ cells were then visualized as Ly6C^hi^ or Ly6C^int^ Ly6G^+^ populations. Percentage and total number of MDSCs was calculated by combining both of these populations. Data is from one experiment and is representative.

Supplemental Figure 2


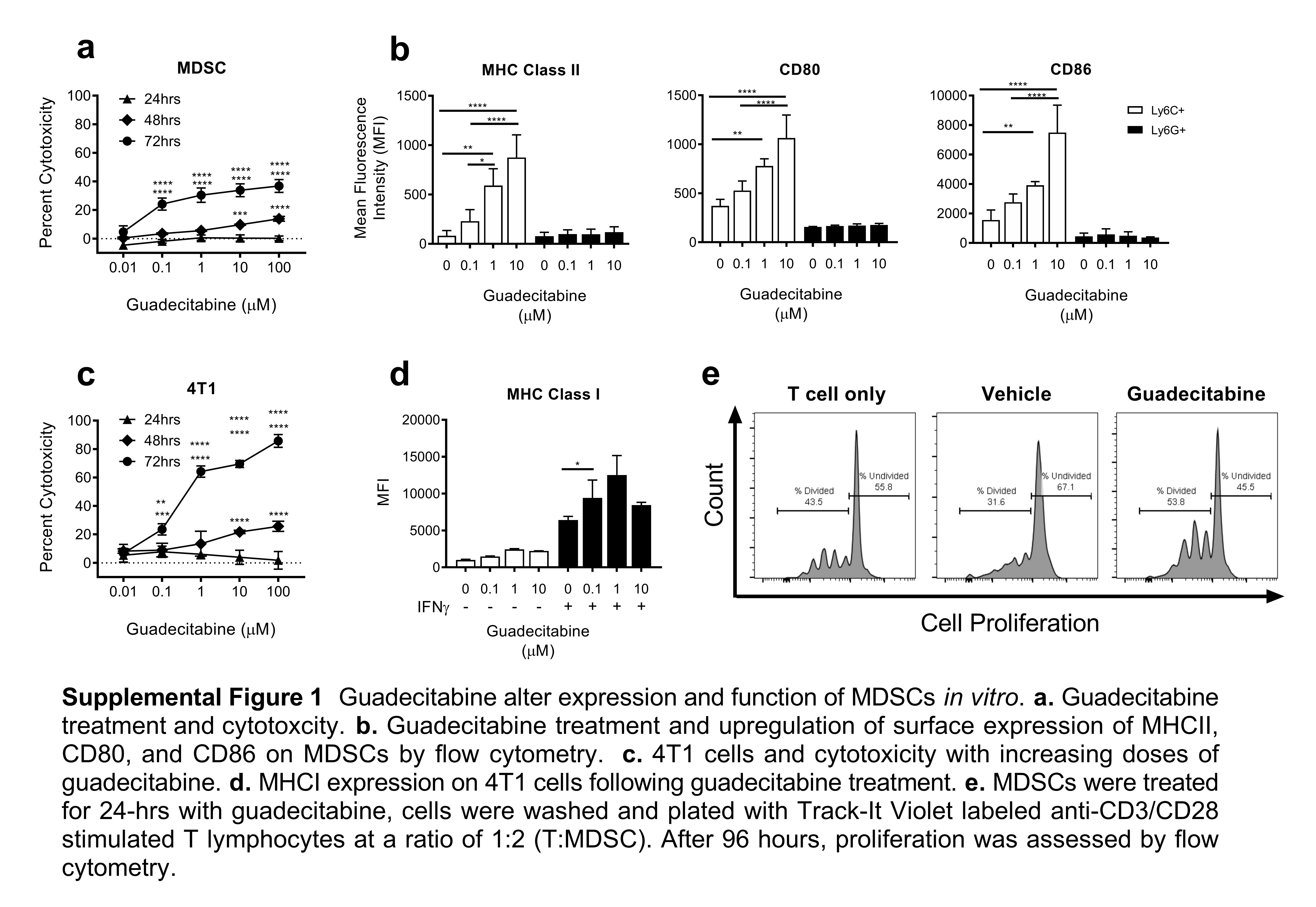


**Supplemental Figure 2**. Guadecitabine alters expression and function of MDSCs *in vitro*. a. MDSC cytotoxicity with increasing doses of guadecitabine over 24hours (triangles), 48hours (diamond), and 72 hours (circles) of treatment. b. Guadecitabine treatment and upregulation of surface expression of MHCII, CD80, and CD80 on MDSCs (Ly6C+; open bars, Ly6G+; closed bars) *in vitro* by flow cytometry. c. 4T1 cytotoxicity with increasing doses of guadecitabine over 24hours (triangles), 48hours (diamond), and 72 hours (circles) of treatment. d. MHCI expression on 4TI cells following guadecitabine treatment for 24 hours ± 5ng/mL IFNγ treatment. e. MDSC activity as measured by T cell suppression after *in vivo* pre-treatment of ADAM10Tg mice with guadecitabine or vehicle. MDSC experiments are n=3 at each data point. 4T1 experiments are n=4 at each data point. Statistical tests were performed by Two-Way ANOVA (a, c). One-Way ANOVA (b, d). For a. and c. top stars apply to comparison between 72 and 48 hours, lower stars apply to comparison between 72 and 24 hours, and the bottom stars apply to comparison between 24 and 48 hours at that particular dosage. e. Representative of n=3. *:p<0.0332; **:p<0.0021 ; ***:p<0.0002; ****:p<0.0001.

Supplemental Figure 3


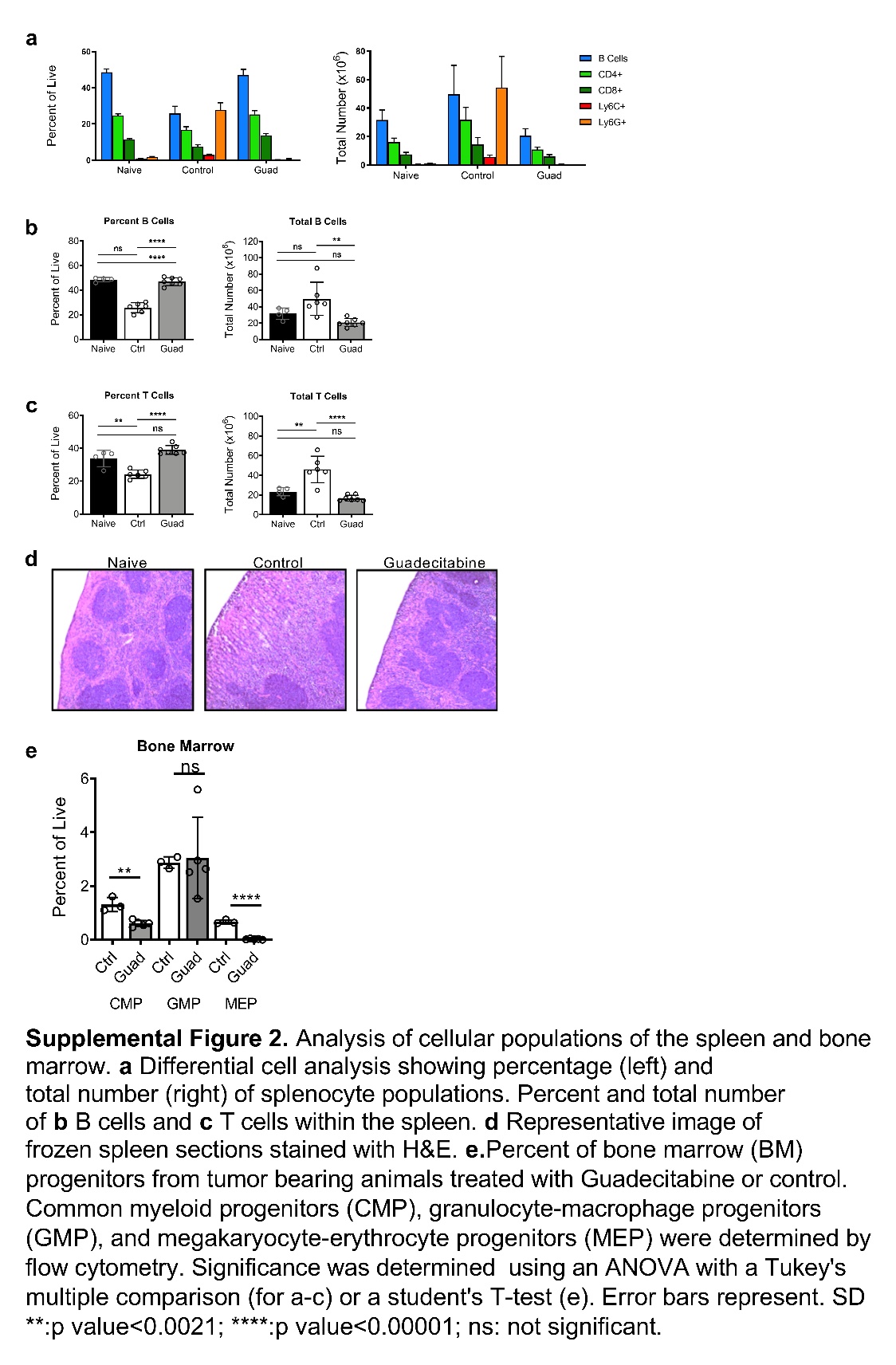


**Supplemental Figure 3.** Analysis of cellular populations of the spleen and bone marrow of tumor-bearing mice with or without guadecitabine treatment. a. Differential cell analysis showing percentage (left) and total number (right) of splenocyte populations. Percent and total number of B cells (b.) and T cells (c.) within the spleen. d. Representative image of frozen spleen sections stained with H&E. e. Percent of bone marrow (BM) progenitors from tumor bearing animals treated with guadecitabine or control were determined by flow cytometery. All were gated on lineage^-^, live, singlets. Common myeloid progenitors (CMP) (IL7Rα^-^,cKit^+^,Sca1^-^,CD16/32^-^,CD34^+^), granulocyte-macrophage progenitors (GMP) (IL7Rα^-^,cKit^+^,Sca1^-^,CD16/32^+^,CD34^+^), and megakaryocyte-erythrocyte progenitors (MEP) (IL7Rα^-^,cKit^+^,Sca1^-^,CD16/32^-^,CD34^-^) were determined. Representative of three experimental replicates, n=4 for naïve, n=6 for control, and n=6 for guadecitabine, (a-d). n=3-5/group for (e). Significance was determined using an ANOVA with a Tukey’s multiple comparison (for a-c) or a student’s T test (e). Error bars represent SD **:p value<0.0021; ****:p value<0.00001; ns:not significant.

Supplemental Figure 4


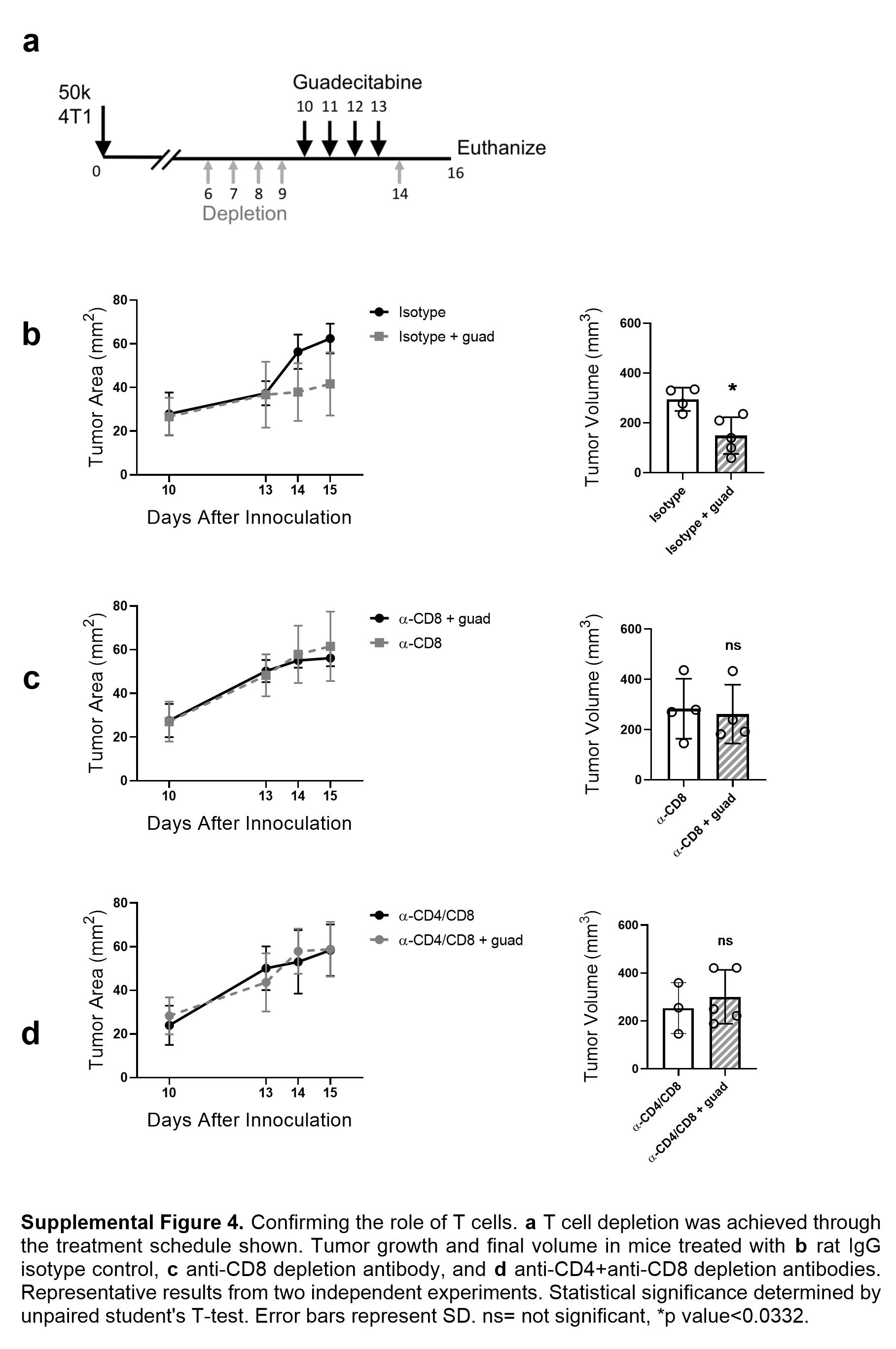


**Supplemental Figure 4**. Confirming the role of T cells. a. T cell depletion treatment schedule. Tumor growth and final volume in mice treated with rat IgG isotype control (b), anti-CD8 depletion antibody (c), and anti-CD4/anti-CD8 depletion antibodies (d). Representative results from two independent experiments. Statistical significance determined by unpaired student’s T-test. Error bars represent SD. ns: not significant; *:p value<0.0332.

Supplemental Figure 5
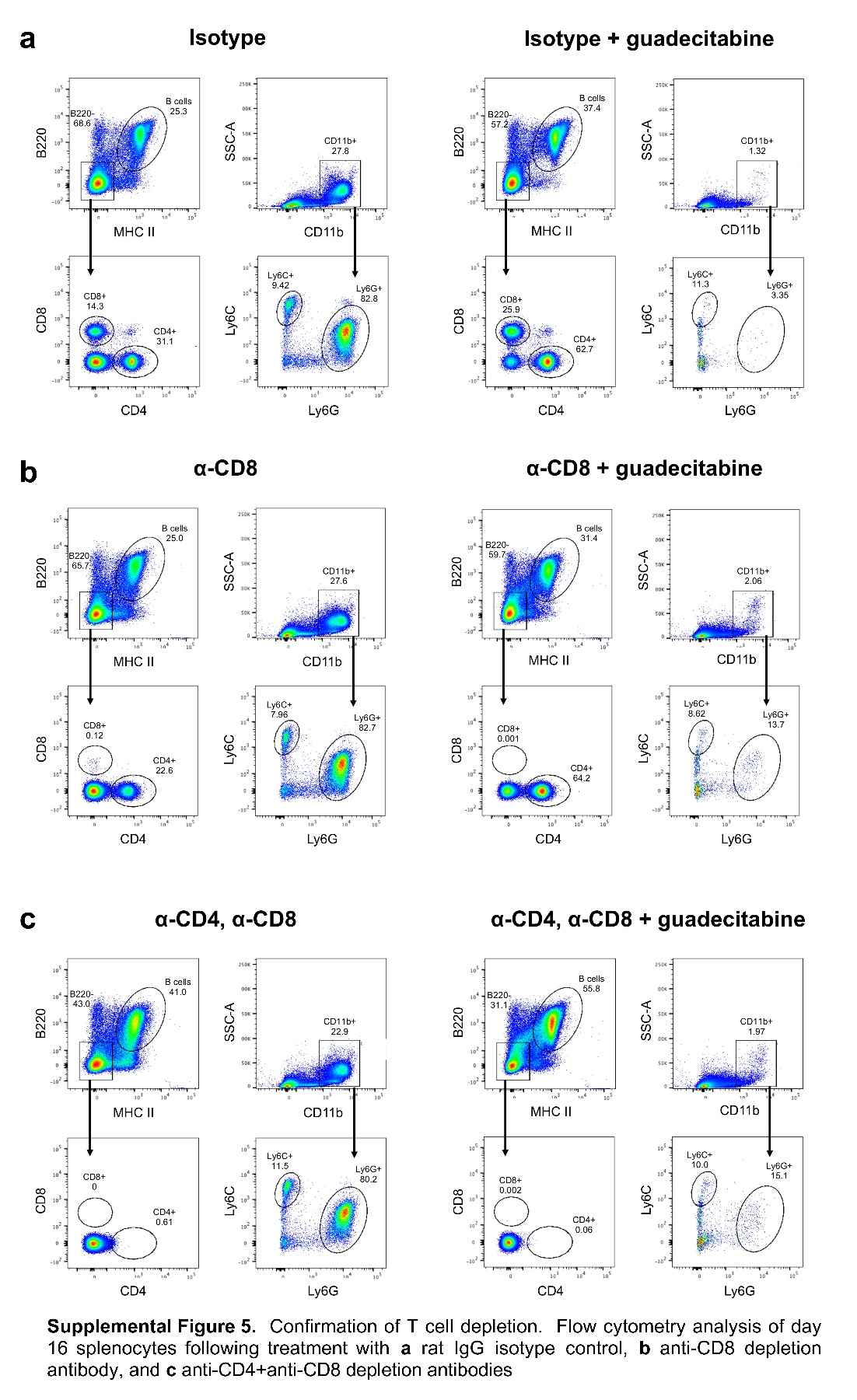


**Supplemental Figure 5.** Confirmation of T cell depletion. Representative flow cytometry analysis of day 16 splenocytes following treatment with rat IgG isotype control (a), anti-CD8 depletion antibody (b), and anti-CD4/anti-CD8 depletion antibodies (c).

Supplemental Figure 6


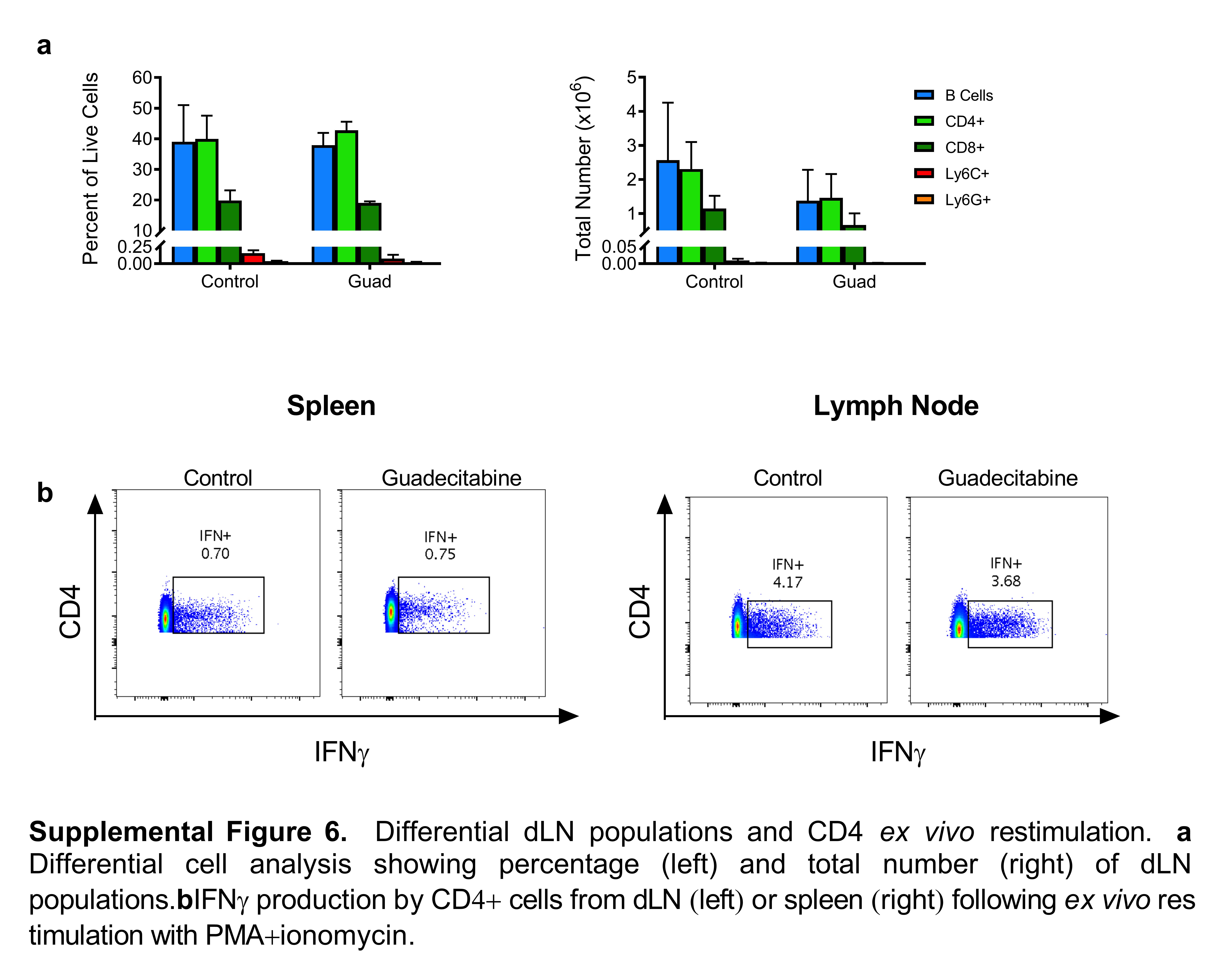


**Supplemental Figure 6**. Differential dLN populations and CD4 ex vivo restimulation. a. Differential cell analysis showing percentage (left) and total number (right) of dLN populations. b. IFNγ production by CD4^+^ cells from dLN (left) or spleen (right) following ex vivo restimulation with PMA and ionomycin. n=3 mice/group for (a). Representative flow cytometry populations in (b).

Supplemental Figure 7





**Supplemental Figure 7.** MDSC activity as measured by Arginase1 staining in the spleen and tumor. Spleens (a) and tumors (b) from D16 were sectioned and stained for Arginase1 (blue), Gr1 (red), and F4/80 (green). Representative images of 4 slides per group.

Supplemental Figure 8


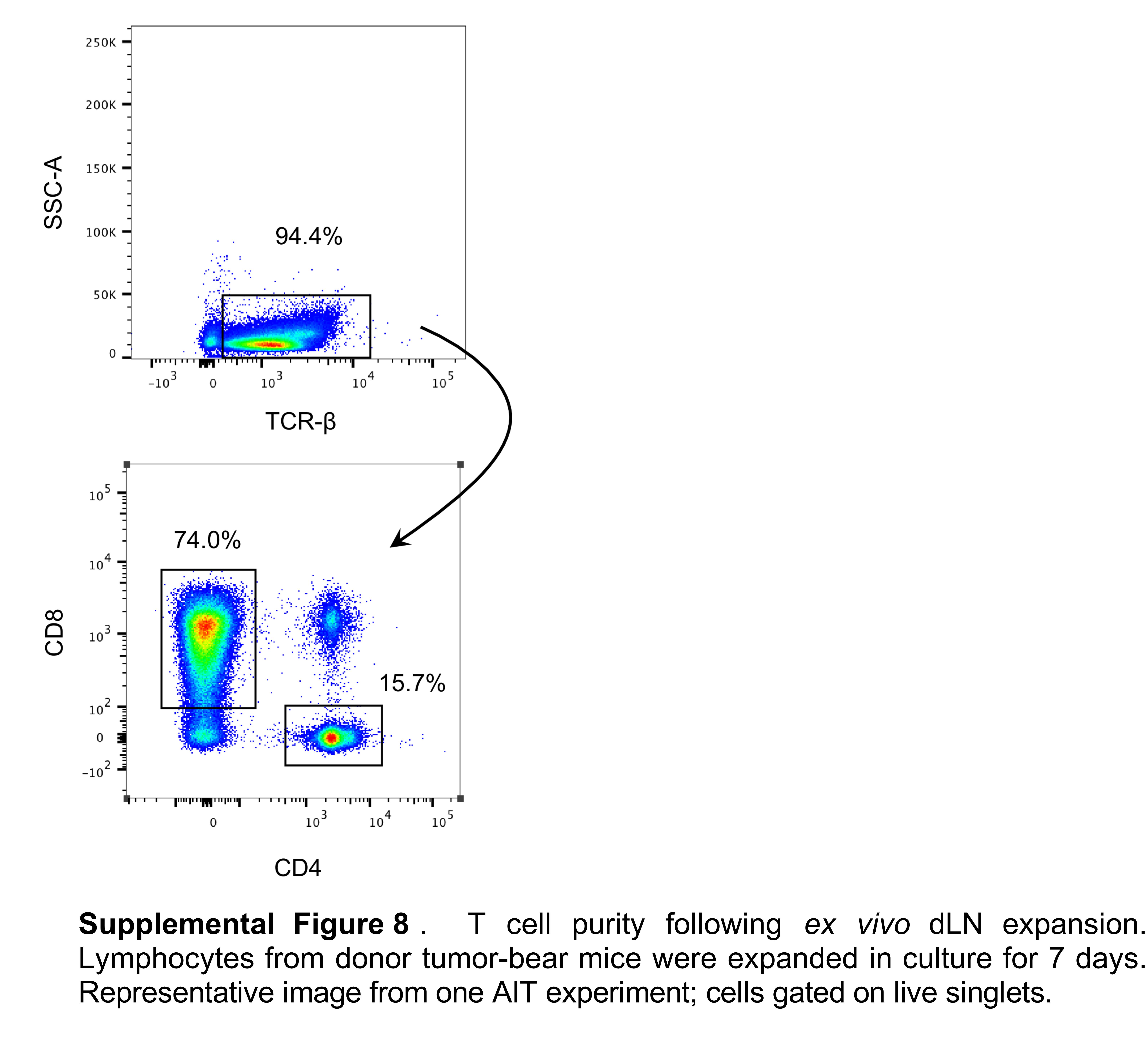

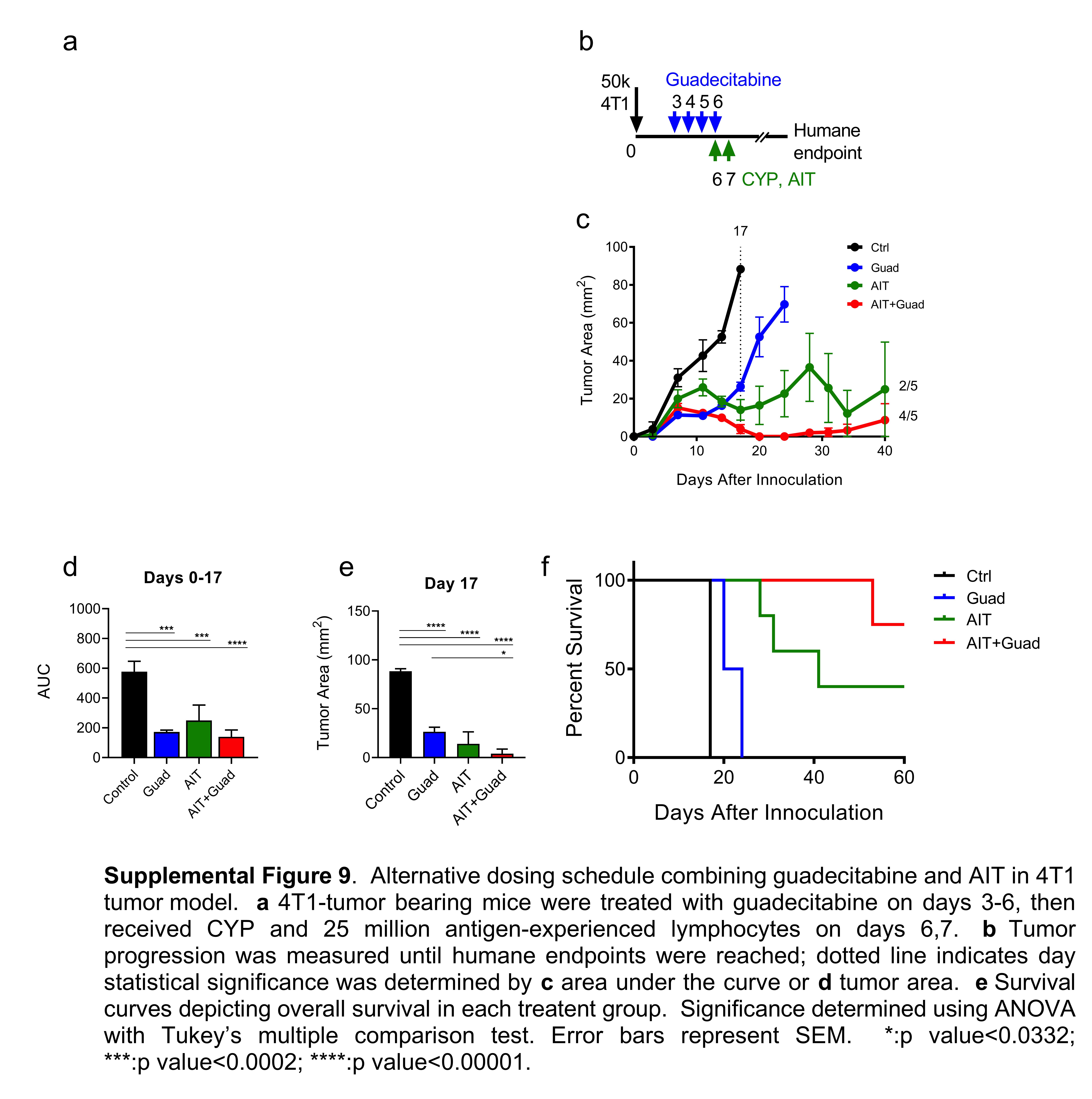


**Supplemental Figure 8.** AIT alternative dosing schedule in 4T1 tumor model. a. T cell purity from 7 day expansion of lymphocytes from dLN of tumor-bearing donor mice. Representative image from one AIT experiment. Gated on live, singlets. b. 4T1-tumor bearing mice were treated with guadecitabine on days 3-6, then received CYP and 25 million antigen-experienced lymphocytes on days 6,7. c. Tumor progression was measured until humane endpoints were researched; dotted line indicates day statistical significance was determined by area under the curve (d) or tumor area (e). f. Survival curves depict overall survival in each treatment group. n>5 mice/group. Significance determined using ANOVA with Tukey’s multiple comparison test. Error bars represent SEM. *:p value<0.0332; ***:p value<0.0002; ****:p value<0.00001.
